# Study of Epigenetic Properties of Poly(HexaMethylene Biguanide) Hydrochloride (PHMB)

**DOI:** 10.3390/ijerph110808069

**Published:** 2014-08-08

**Authors:** Edmond E. Creppy, Aboudoulatif Diallo, Serge Moukha, Christophe Eklu-Gadegbeku, Daniel Cros

**Affiliations:** 1 Department of Toxicology, University Bordeaux Segalen, 143, rue Léo Sagnat 33076-Bordeaux, France; E-Mails: aboudoulatif@yahoo.fr (A.D.); serge.moukha@u-bordeaux2.fr (S.M.); chriskeklugadegbeku@gmail.com (C.E.-G.); 2 Laboratoire PAREVA, Z.I. du bois de LEUZE 25, avenue Marie Curie 13310-Saint-Martin de CRAU, France; E-Mail: d.cros@pareva.fr

**Keywords:** Poly(Hexamethylene Biguanide) hydrochloride (PHMB), epigenetic effects, cytokines production

## Abstract

Poly(HexaMethylene Biguanide) hydrochloride (PHMB) **CAS No. [32289-58-0]** is a particularly effective member of the biguanides antiseptic chemical group, and has been in use since the early fifties in numerous applications. It has been proposed that PHMB be classified as a category 3 carcinogen although PHMB is not genotoxic. It has been hypothesized that PHMB may have epigenetic properties effects, including non-genotoxic modifications of DNA bases, DNA methylation and mitogenic cytokine production. These properties have been assessed in vitro using 3 cell types: Caco-2 cells (from a human colon adenocarcinoma) with a non-functional *p53* gene. (∆p53: mut p53), N2-A (Neuro-2A cells, mouse neural cells), the brain being a possible target organ in rodents and HepG2 cells (human hepatocellular carcinoma) with functional *p53* gene. From the concentration 1 μg/mL up to 20 μg/mL of PHMB, no effect was observed, either growth stimulation or inhibition. Viability testing using neutral red led to an IC 50 of 20–25 μg/mL after treatment with PHMB for 3 h, whereas the MTT test led to IC50 values of 80 μg/mL, 160 μg/mL and 160 μg/mL respectively for HepG2 cells, Neuro-2A cells and Caco-2 cells. PHMB does not induce significant oxidative stress (production of MDA or lipoperoxidation, nor does it induce hydroxylation of DNA (8-OH-dG) and/or its hypermethylation (m5dC), the latter being strongly implicated in DNA replication and regulation and cell division. PHMB does not induce significant production of mitogenic cytokines such as TNF-α (tumor necrosis factor), interleukins (IL-1 alpha), and the transcription factor nuclear factor kappa B (NF-κB) which can cause either apoptosis or stimulate the growth of transformed cells or tumors. Instead, from concentrations of 20 to 100 μg/mL, PHMB kills cells of all types in less than 3 h. The expression of genes involved in the mechanisms of cell death induced by PHMB, including *p53*, the pro apoptotic gene *bax* and others, the anti-apoptotic *bcl-2* and caspase-3 has been evaluated by RT-PCR. Finally, the status of GAP-junctions (GJIC) in the presence of PHMB has been determined and appeared to not be significantly affected. Taken together the data show that in vitro PHMB does not exhibit clear and remarkable epigenetic properties except a slight increase of some cytokines and transcription factor at higher concentrations at which cell lysis occurs rapidly.

## 1. Introduction

Poly (HexaMethylene Biguanide) hydrochloride (PHMB) (CAS No. 32289-58-0, previously 91403-50-8) belongs to the chemical family of antiseptic biguanides. It has been used since the early nineteen-fifties and has been found to be particularly effective as a hard surface disinfectant, water treatment agent (algaecide and sanitizer, in swimming pools, SPAs and water tanks), preservative agent in several products due to its bacteriostatic or bactericidal properties in a large number of industrial processes and water systems, and as a personal care products preservative. PHMB is effective against pathogens such as bacteria, amoebae and yeast [1,2,3,4]. Additionally there is an increasing number of reports showing that PHMB has anti-HIV activity [5,6,7]. More interestingly it is active over a wide range of pH (at least from pH 3 to 10), and is temperature (no decomposition until 200–220 °C) and light (including UV) stable. PHMB does not generate chlorinated by-products (CBPs) or trihalomethanes (THMs) found in chlorine-based treatments. For general water treatment, the recommended dose is 20 to 40 mg/L of a 20% PHMB solution (4 to 8 mg/L of active substance) (US-EPA, 2004) [8]. Compared to chlorine based products, PHMB is not irritating to the eyes or skin, at the recommended doses used in swimming pools.and even in cases of accidental exposure where doses reached 50 and 70 mg/L (10 and 14 mg/L of active substance, respectively) no toxicity was observed [8].

According to the Biocidal Products Directive (Directive 98/8/EC, also known as BPD), PHMB has been classified as a suspected carcinogen in rodents, based upon three studies conducted during the review of the BPD dossier [8,9,10,11,12]. This assessment led to a classification of PHMB as a Carc cat. 2 + H351 (Suspected of causing cancer), category 3; R40 (Limited evidence of carcinogenicity) under the Directive 67/548/EC of the European Parliament and of the Council of 16 February 1998 [13]. 

PHMB is not genotoxic [8,10,11,12], so the present classification raises questions as to possible mechanisms for the rodent carcinogenesis of PHMB. The present experiments are therefore designed to confirm the nongenotoxicity of PHMB and assess its epigenetic effects [14,15,16,17,18] including possible nongenotoxic DNA base modifications [16,17,18,19,20], mitogenic cytokine production and integrity of the gap junction intercellular communication [21,22] using 3 cell types: (1) Caco-2 cells (from a human colon adenocarcinoma) with a non-functional *p53* gene (∆p53: mut p53), (2) N2-A (Neuro-2A cells, mouse neural cells), the brain being a possible target organ in rodents and (3) HepG2 cells (human hepatocellular carcinoma) with functional *p53* gene [23,24].

## 2. Materials and Methods

### 2.1. Chemicals—Poly(HexaMethylene Biguanide) Hydrochloride, PHMB (CAS n° 32289-58-0, Previously 91403-50-8)

**Scheme 1:**
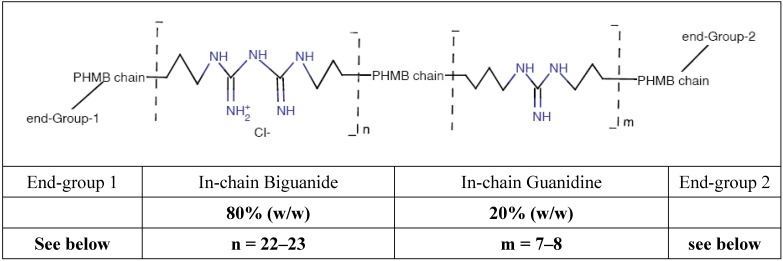
Structure of *Poly(HexaMethylene Biguanide) hydrochloride*, PHMB (CAS n° 32289-58-0, previously 91403-50-8), showing structural functions.

**Scheme 2:**
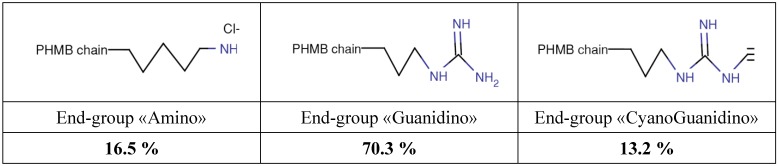
Structure of end-groups, Amino, Guanidino and CyanoGuanidino of *Poly(HexaMethylene Biguanide) hydrochloride*, PHMB (CAS n° 32289-58-0, previously 91403-50-8).

PHMB is a limpid, slightly opalescent water solution which is manufactured and commercialized as the hydrochloride (pH = 4 to 5), at a concentration of 20% (w/w). For these studies, only PHMB P100, the powder of the active ingredient, was used which was obtained by freeze-drying the standard 20% solution. The purity of the PHMB was 99.54 ± 0.09%. The mean characteristics of the PHMB used (Batch ref. #8825, Laboratoire Paréva, Saint-Martin de Crau, France) were Mn = 1415 g/mol; Mw = 6630 g/mol. The oligomer structure is shown in the generic formula below (Scheme 1):

There three end-groups were amino, guanidino and cyanoguanidino at concentrations of 16.5, 70.3 and 13.2%, respectively (Scheme 2).

Stock solutions were prepared and serially diluted to obtain the required treatment concentrations.

### 2.2. Cell Lines

#### 2.2.1. Neuro-2A Cells Were Derived from a Mouse Neuroblastoma and Have a Functional *p53* Gene

They were cultured in Roswell Park Memorial Institute (RPMI) medium (Vendor/location) containing 10% fetal calf serum, L-glutamine, sodium pyruvate, and 1% penicillin-streptomycin.

#### 2.2.2. HepG2 Cells Were Derived from a Human Hepatocellular Carcinoma and Have a Functional *p53* Gene

Cells were cultured in Minimal Essential Medium (MEM), (Sigma, France) containing Glutamax (Sigma), 10% fetal calf serum (Sigma), 1% nonessential amino acids, 100 IU/mL penicillin, and (100 mg/mL) streptomycin (Sigma) in 96 well plates.

#### 2.2.3. Caco-2 Cells Were Derived from a Human Colon Adenocarcinoma and Lack Expression of *p53*

Cells were cultured in Dulbecco’s Modified Eagle’s Medium, (DMEM) containing 10% FCS, L-glutamine, 1% penicillin-streptomycin, and 1% Amphothericin B (1%).

All cells were grown at 37 °C in a mixture of air (95%) and CO_2_ (5%). Experiments were repeated at least six times using 8 wells per concentration. Negative controls were made with culture medium without PHMB and positive controls with either cadmium chloride (CdCl_2_, 2 mg/mL) or hydrogen peroxide (2 mM).

### 2.3. Cell Viability Tests

#### 2.3.1. MTT (3-(4,5-Dimethylthiazol-2-yl)-2,5-Diphenyltetrazolium Bromide) Test

This test is an indicator of mitochondrial activity. The principle is as follows: the action of succinate dehydrogenase (SDH) in viable cells converts succinate into fumarate. At the same time, the coenzyme FAD^+^ is converted to FADH_s_ resulting in the reduction of the yellow MTT to the insoluble blue colored product, formazan. For this, cells were cultured in 96-well plates 24 h prior to beginning treatment. After incubation for 1, 3 or 24 h with PHMB, the culture medium was removed by inverting the plates and replaced by 100 μL of MTT (0.5 mg/mL). The cells were incubated for two hours, then a solution of 100 μL of DMSO was added (cell lysis and dissolution of formazan). After 45 min of vortexing, the absorbance was read at 560 nm using a plate reader. A minimum of six wells, was used for each concentration of PHMB.

#### 2.3.2. Neutral Red Test

This test is performed according to the protocol described by Yusup *et al.* [25]. The principle of this protocol is based on the integrity of the cellular, lysosomal and endosomal membranes. The neutral red penetrates inside living cells and colors the cytoplasm of still viable cells. This red color of neutral red is released and quantified by measuring the optical density. Briefly, cells were seeded in 96-well microplates (10,000 cells/200 μL/well) and incubated with PHMB. After incubating for 1, 3 or 24 h, the medium with or without PHMB were removed by inverting the plates and replaced by 200 μL of a solution of neutral red (50 mg/mL) freshly prepared. The cells were then re-incubated for 4 h at 37°C, rinsed with 200 μL of PBS (to remove extracellular neutral red) and lysed with 200 μL of an acetic acid/50% ethanol solution (1:99, V/V). The absorbance was determined at 540 nm after 15 min of stirring.

### 2.4. Evaluation of Lipid Peroxidation

The determination of MDA (malondialdehyde) is probably the most widely used technique for evaluation *in vitro* of the oxidation of lipids and peroxidation. Caco-2, HepG2 or Neuro-2A cells were seeded (1.5 × 10^5^ cells/mL complete medium) in 24-well plates, and after 24 h of preincubation, the PHMB was added to the culture medium at concentrations of 20, 40 and 80 μg/mL. The cells were incubated with PHMB for 1, 3 h or 24 h at 37°C. Extraction and determination of MDA-thiobarbituric acid (TBA) adduct by HPLC was performed as described by Ennamany *et al.* [26]. Briefly, after 24 h of preincubation with or without PHMB or positive control, cells were collected using 0.05% trypsin with 0.02% ethylenediamine tetracetic acid (EDTA) (Sigma-Aldrich) and centrifuged at 600 g for 10 min at 4°C. As the amount of MDA measured is presented relative to the protein content of cellular homogenates, 10 μL of each cell lysate was removed prior to processing for determination of DMA-TBA adducts for determination of protein concentration using the colorimetric method of Bradford (1976) [27].

### 2.5. Determination of Levels of 8-Hydroxydeoxyguanosine (8-OHdG) and of 5-Methyldeoxycytosine (m5dC)

The cells were seeded in 25 cm^2^ dishes at a concentration of 10^6^ cells/mL. After 3, 12 or 24 h incubation at 37 °C, cells were treated with 20, 40 or 80 μg/mL PHMB. Control wells were treated with similar volumes of ultrapure water (<1% v/v) in culture medium. The DNA extraction was performed using the Wizard Kit (Promega, France). The cells were lysed and RNA was hydrolyzed by RNase followed by precipitation of proteins. DNA was precipitated by alcohol. Optical density at 260 nm (OD_260_) was used to determine DNA quantity and purity was determined by the OD_260_/OD_280_ ratio. The enzymatic hydrolysis of DNA was performed using the method described by Matias *et al.* [28]. Briefly, the DNA sample (10 μg total/ test) was hydrolyzed to produce 2’-deoxymononucleosides, using DNase I, Nuclease P1, (from Sigma) followed by alkaline phosphatase treatment. To quantify m5dC and 08-OHdG, the nucleoside mixture obtained was analyzed by HPLC with a dual electrochemical detector (EC) for detection of 08-OHdG and a UV detector for detection of m5dC and the four normal bases [20,26]. The chromatographic data were confirmed by ELISA (competitive method) using Oxiselect Kit (Cell BioLabs, San Diego, USA) or Global DNA Methylation ELISA kit (Cell Biolabs, Mondolsheim, France ) respectively.

### 2.6. Evaluation of Apoptosis by Measuring the Activity of Caspase-3

The cells were seeded into 25 cm^2^ dishes at a concentration of 10^6^ cells/mL. After 24 h incubation at 37°C, the cells were treated with cytotoxic concentrations of PHMB (20–200 or 500 μg/mL) for 3, 6 and 24 h. Controls were treated with ultrapure water (<1% v/v) at similar volumes in the culture medium. After treatment, cells were lysed for determination of caspase 3 activity using the CaspACE^TM^ Assay System, Colorimetric (Promega). The enzyme caspase-3 (DEVDase) cleaves its substrate Ac-DEVD-NAPL and Ac-DEVD-pNA labelled with the chromatophore p-nitroaniline (pNA). The yellow color produced is measured at 405 nm and is proportional to the presence of free pNA chromatophore and therefore, the activity of the enzyme caspase-3. The specific activity (SA) of caspase-3 (SA) was determined as follows: 

SA = pmol pNA liberated/hour/mg of total protein.

### 2.7. Evaluation of the Expression of Genes Involved in the Mechanisms of Cell, Cycle, Cell Death and Inhibition of Gap Junctional Intercellular Communication (GJIC)

The cells were seeded and treated in the same manner as above. Total RNA was extracted from approximately five million cells, using the kit (Promega). The expression levels of *p21*, *p53*, *bax*, *bcl-2*, and connexion (*cx43*) were determined by RT-PCR. The complementary DNA (cDNA) was synthesized using 1 μg of total RNA, a reverse transcriptase and primers “Static Random Primers”, Promega kit, Saint Quentin en Yvelines, France (Table 1) Ten μL of cDNA was used per PCR reaction. cDNA was amplified using the following program: 25 cycles for β-actin, 35 cycles genes of interest, denaturation at 94 °C for 30 s), annealing at 55 °C for 30 s and elongation at 68 °C for 2 min). Reaction was terminated by incubating 5 min at 98 °C and 5 min at 4 °C. The constitutively expressed GAPDH gene was used as a housekeeping gene [29]. The result of amplification reactions was assessed on 2% agarose gel.

**Table 1:** Sequences of oligonucleotides used in amplification of genes of interest.

Oligonucléotides	Séquences oligonucléotides	Tm
Bactf	AAgACCTgTACgCCAACACAg	60.22
Bactr	ATACTCCTgCTTgCTgATCC	59.85
BAXf	ACCAAgAAgCTgAgCgAgTg	60.73
BAXr	ggAggAAgTCCAATgTCCAg	59.51
BCL2f	ggATTgTggCCTTCTTTgAg	59.67
BCL2r	TCACTTgTggCCCAgATAgg	61.05
P53f	ATAgTgTggTggTgCCCTATg	59.75
P53r	TCATTCAgCTCTCggAACATC	60.36
P21f	TggAACTTCgACTTTgTCACC	60.14
P21r	AAATCTgTCATgCTggTCTgC	60.28
Cx43f	GAATCCTGCTCCTGGAGC	61.05
Cx43r	GATGCTGATGATGTAGCAT	60.36

### 2.8. Statistical Analysis

Statistical analyzes of the values were performed using GraphPad Prism software version 5.04 for Windows (GraphPad Software, San Diego, CA, USA). Five criterion were used to evaluate cell culture runs for acceptance. The culture medium amended with medium (without supplementation) did not exceed 5% of culture volume and did not induce more than 05% cytotoxicity. (i) In a given plate, the %CV of the mean measured values had to be ≥20 % compared to the control with medium and cells (100% viability) to be considered as positive. (ii) The mean of controls (with or without vehicle) for intra-plate and inter-plate measurements at the various incubation times could not differ by more than 15%. (iii) The normality of the population was tested with the D’Agostino test. Comparison of Gaussian populations was performed using the Student t test (significant values for *p* < 0.05 CI = 95% and *p* < 0.001 CI = 99%). If distribution was not normal, the Mann-Whitney non-parametric test was used. (iv) The dose-response curves were fitted using a four-parameter logistic model (4PL) (Hill slope model). (v) The fitted dose-response curves had to have a R^2^ > 0.85.

## 3. Results

### 3.1. Effect of PHMB on Cellular Viability

#### 3.1.1 Cellular Viability Measured by the MTT Test

**Figure 1:**
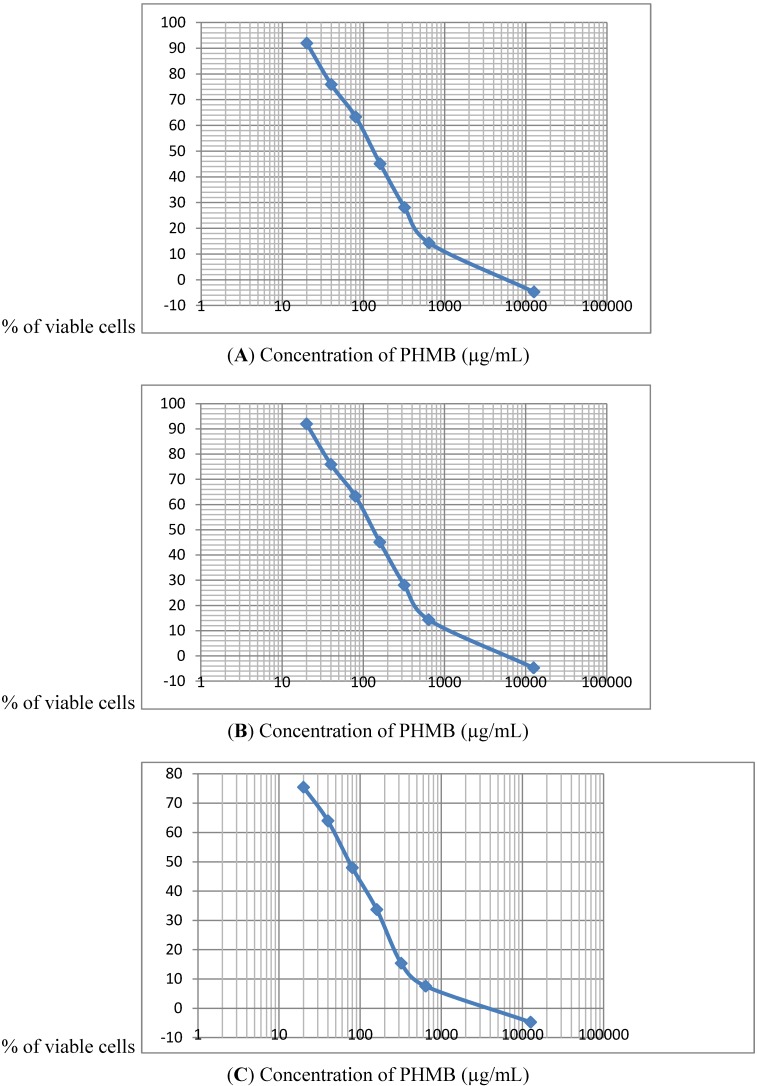
IC50% as measured by MTT test following 3h of incubation with PHMB for (**A**) Neuro-2A, 160 μg/mL; (**B**) Caco-2, (**C**) HepG2 cells, 80 μg/mL.

The IC50% values measured by the MTT test following 3 h of incubation with PHMB, for Caco-2 cells and for Neuro-2A cells was 160 μg/mL. However, the IC50% for HepG2 cells of 80 μg/mL was clearly lower (Figure 1A–C).

#### 3.1.2. Cellular Viability Measured by the Neutral Red Test

The IC50% values, measured by the neutral red test after 1, 3 and 24 h of incubation with PHMB, were similar for the three cell lines, and much lower than the LC50% values measured by the MTT test (Table 2).

**Table 2:** Evaluation of cellular viability using the neutral red test, following 1, 3 and 24 h of incubation with PHMB in the three selected cell lines.

IC 50% (μg/mL)/Time of incubation (h)	IC50% of PHMB in different cell lines (μg/mL)		
	Caco-2	HepG2	Neuro-2A
**IC 50% 1 h**	10 ± 2	12 ± 1	8 ± 2
**IC 50% 3 h**	20 ± 2	25 ± 5	25 ± 5
**IC50% 24 h**	25 ± 10	35 ± 10	35 ± 10

### 3.2. Cytotoxicity of PHMB on Caco-2, Neuro-2A and HepG2 Cells

**Figure 2:**
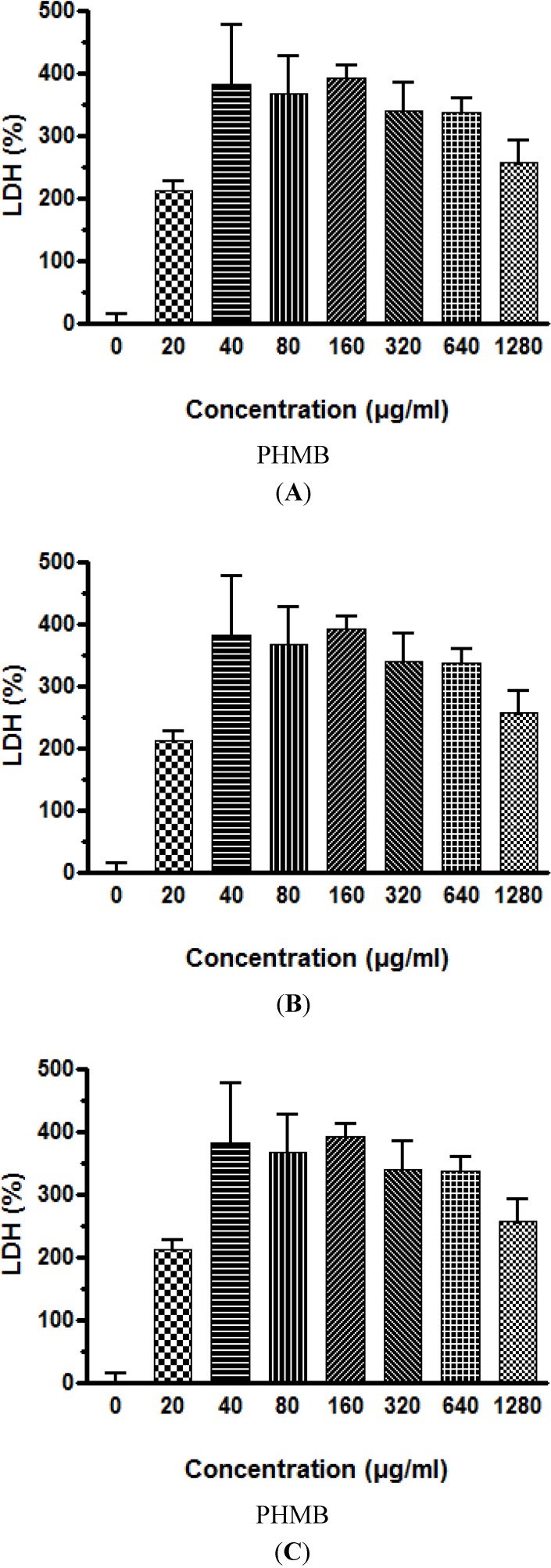
Effect of incubation with PHMB for 3h on the leakage of LDH into the culture medium of (**A**) Caco-2 cells; (**B**) Neuro-2A cells; and (**C**) HepG2 cells.

Following viability testing, direct cytotoxicity of PHMB on the selected cell lines was evaluated. The leakage of LDH into the culture medium of the three cell lines following incubation with increasing concentrations of PHMB was similar (Figure 2A–C). LDH leakage in the culture medium increased rapidly to 200% at PHMB concentration of 20 μg/mL and reached a maximum of 400% at 40 to 80 μg/mL.

### 3.3. Effects of PHMB on the Integrity of DNA

#### Effect on DNA Fragmentation

**Figure 3:**
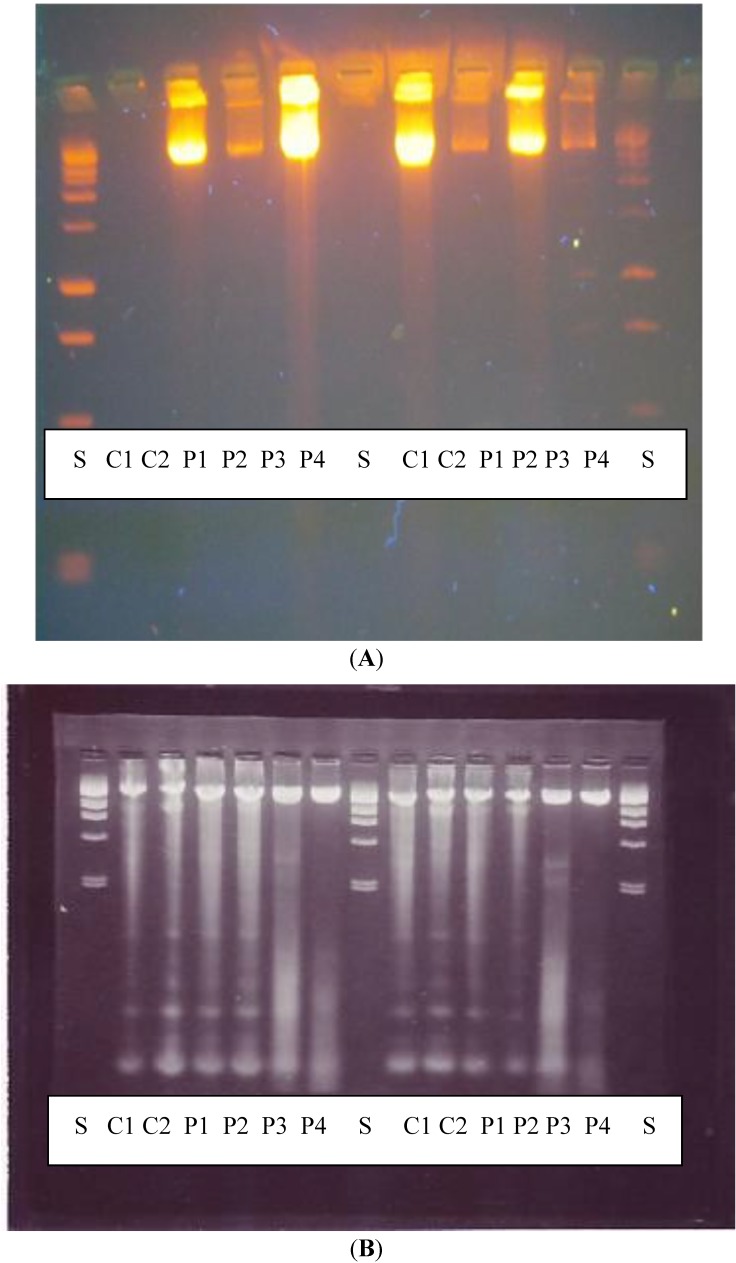
Effects of PHMB on DNA fragmentation using 1% agarose gel electrophoresis in (**A**) HepG2 cells; (**B**) Neuro-2A cells.

The effect of PHMB on the integrity of DNA was evaluated by agarose gel electrophoresis using two different methodologies, direct gel (2% agarose) electrophoresis on extracted DNA [22,23] and the Comet test. Treatment with PHMB did not cause an increase in DNA fragmentation at any concentration tested when compared to the control cells (Figure 3A,B and Table 3), thus confirming that PHMB is not genotoxic.

**Table 3:** Percentage of DNA in the comet tail (comet assay).

Concentration (μg/mL or mM)	% of DNA in the tail		
	Caco-2	HepG2	Neuro-2A
PHMB 1 μg/mL	8 ± 5	7 ± 5	8 ± 3
PHMB 5 μg/mL	8 ± 2	8 ± 2	8 ± 3
PHMB 10 μg/mL	10 ± 3	10 ± 3	8 ± 3
PHMB 20 μg/mL	8 ± 2	8 ± 3	10 ± 2
PHMB 30 μg/mL	10 ± 4	10 ± 2	10 ± 4
PHMB 40 μg/mL	8 ± 3	10 ± 3	10 ± 2
PHMB 80 μg/mL	10 ± 3	10 ± 5	10 ± 5
Positive control (H_2_O_2_, 10 mM)	60 ± 5	66 ± 5	65 ± 10
Negative control (0 μg/mL PHMB)	6 ± 2	7 ± 2	8 ± 5

S = Sizing marker; (bp from 100 to 2200 down-up); C = Control; P = PHMB; P1 and P2, DNA of Neuro-2A cells, following incubation for 30 min with 30 μg/mL PHMB; P3 and P4, DNA of Neuro-2A cells following incubation for 30 min with 100 μg/mL PHMB.

### 3.4. Mechanism of Cell Death Induced by PHMB

#### 3.4.1. Evaluation of Oxidative Stress by Determination of MDA

The effect of PHMB (20–80 μg/mL) on MDA production (pmol/mg of protein) in Neuro-2A, Caco-2 and HepG2 cells was evaluated and compared to H_2_O_2_ (positive control) following 3 h of incubation. MDA production in negative control cells was 70 ± 25 pmol/mg of protein. The production of MDA in the presence of PHMB (20 to 80 μg/mL) in all 3 cell lines was similar to production in the negative control (Table 4) indicating PHMB did not induce MDA production. There is no time or concentration dependency observed in MDA production in the three cell lines.

**Table 4:** Effect of PHMB (20–80 μg/mL) on MDA production (pmol/mg of protein) in Neuro-2A, Caco-2 and HepG2 cell lines. Cells were incubated with PHMB or H_2_O_2_ (positive control) for 3 h, 12 h and 24 h.

Cell line and Incubation time Neuro-2A	PHMB 20 μg/mL	PHMB 40 μg/mL	PHMB 80 μg/mL	H_2_O_2 _(10 mM)
**3h**	82.60	120.7	25.21	161.03
**12h**	39.42	68.92	37.16	447.15
**24h**	60.77	85.00	28.68	520.32
**Mean** **±**	60.93 **	91.54 *	30.35 **	**376.20**
**SD**	**12.47**	**15.30**	**3.55**	**109.60**
**Caco-2**	**PHMB 20 μg/mL**	**PHMB 40 μg/mL**	**PHMB 80 μg/mL**	**H_2_O_2 _(10 mM)**
**3h**	55.59	15.64	68.36	236.89
**12h**	53.54	15.06	69.87	443.38
**24h**	62.33	11.27	56.70	156.88
**Mean** **±**	57.15 **	13.99 **	64.98 **	**279.1**
**SD**	**2.65**	**1.37**	**4.16**	**85.35**
**HepG2**	**PHMB 20 μg/mL**	**PHMB 40 μg/mL**	**PHMB 80 μg/mL**	**H_2_O_2 _(10 mM)**
**3h**	65.90	25.60	78.60	236.89
**12h**	63.40	25.76	79.80	443.38
**24h**	72.30	21.70	66.70	156.88
**Mean** **±**	67.15 **	18.66 **	74.71 **	**279.1**
**SD**	**7.65**	**6.7**	**9.20**	**85.35**

* *p* < 0.05; H_2_O_2_ *vs.* PHMB; ** *p* < 0.01; H_2_O_2_ *vs.* PHMB.

#### 3.4.2. Oxidative Modification in DNA

The effect of PHMB on DNA bases oxidation in the three different cell lines, Neuro-2A, Caco-2 and HepG2 was evaluated. Cells were incubated with PHMB or cadmium (Cd^2^+) (positive control) for 3 h or 24 h. The negative control was culture medium without PHMB. Results are expressed as ng of 8-OHdG/mL for 10 μg of DNA. Relative values were calculated as oxidized bases in the DNA of PHMB-treated cells/oxidized bases in the DNA of untreated cells). When normalized to the concentration of 8-OHdG in the negative control, a slight increase in 8-OHdG in the DNA of PHMB-treated cells was observed, however the increase was not concentration-dependent (Table 5).

**Table 5:** Effect of PHMB on DNA base oxidation in Neuro-2A, Caco-2 and HepG2 cell lines. Cells were incubated with PHMB or cadmium (Cd^2^+) (positive control) for 3 h or 24 h. Results are expressed as ng of 8-OHdG/mL in 10 μg of DNA. Relative values were calculated as oxidized bases in the DNA of treated cells/oxidized bases in the DNA of untreated cells.

Cell Lines	Control and mean	PHMB 40 μg/mL	PHMB 80 μg/mL	Cadmium
	absolute value of 8-OHdG	Relative value ± SEM	Relative value ± SEM	Relative Value ± SEM
Neuro-2A	2.1	1.29 **	1.02 **	11.87
	-	0.20	0.08	0.08
Caco-2	2.4	0.92 **	0.98 **	12.80
	-	0.09	0.10	0.10
HepG2	2.7	1.31 **	1.39 **	15.80
	-	0.18	0.21	0.10

** *p* < 0.01; Cd^2+^ *vs.* PHMB. 8-OHdG was first separated by HPLC and quantified by electrochemical detection. The results were confirmed by ELISA (competitive method) using Oxiselect kit. Only chemical data are presented here.

### 3.5. DNA Methylation Following Exposure to PHMB

DNA methylation is involved in the regulation of DNA replication, cell division, oncogene activation and/or repression of tumor suppressing genes [20,30,31,32]. The DNA methylation rate was low and variable according to the cell line, but not related to the concentration of PHMB in the medium. However, the rate is increased by 33 to 50% in the presence of PHMB for 24 h (Table 6). The lowest rate of hypermethylation was found in the Neuro-2A cells and the highest in HepG2 cells.

**Table 6:** Effect of PHMB on DNA cytosine methylation in Neuro-2A, Caco-2 and HepG2 cells. Cells were incubated with PHMB or cadmium (Cd^2^+) (positive control) for 3 h or 24 h. Results are expressed as m5dC nmol/mL for 10 μg of DNA. Relative values were calculated as % of m5dC in the total dC in the DNA of treated cells/% of m5dC in the total dC in the DNA of untreated cells).

Cells	Control value for 10 μg of DNA/mL	PHMB 40 μg/mL	PHMB 80 μg/mL	Cadmium
		Relative value ± SEM	Relative value ± SEM	Relative Value ± SEM
Neuro-2A	3.4.	1.33 **	1.066 **	16.066
	-	0.35	0.33	0.40
Caco-2	2.7-	1.36 **	1.45 **	17.36
	-	0.20	0.18	0.64
HepG2	2.4-	1.33 **	1.50 **	15.05
	-	0.22	0.27	0.45

** *p* < 0.01; Cd^2+^ *vs.* PHMB. m5dC was first separated by HPLC and quantified by UV detection at 254 nm. The results were confirmed by ELISA (competitive method) using Global DNA Methylation ELISA kit (Cell Biolabs.UK). Chemical data are presented here.

### 3.6. Mechanism of Cell Death in the Presence of PHMB

#### Effect of PHMB on Caspase-3 Activation

Effect of PHMB on caspase-3 activation in the three different cell lines Neuro-2A, Caco-2 and HepG2. The cells were incubated with PHMB or cadmium (Cd^2+^) (positive control) for 3 h and 24 h. Treatment with PHMB did not activate caspase-3 in any of the cell lines tested. A slight activation observed at 80 μg/mL is not significant, because of the large variation in results at that concentration (9.06 ± 6.2, 6.65 ± 5.2 and 3.06 ± 3.2, respectively for Neuro-2A, Caco-2 andHepG2). Caspase-3 activation at 40 μg/mL PHMB was similar to the negative control (Table 7).

**Table 7:** Effect of PHMB on caspase-3 activation in Neuro-2A, Caco-2 and HepG2 cells. Cells are incubated with PHMB or cadmium (Cd^2+^) (positive control) for 3 h and 24 h. Caspase-3 activity was measured using Promega CaspACE^TM^ Assay System colorimetric kit. Results are expressed as pmol/μg of protein/h.

Cell Line Neuro-2A cells	Control	PHMB 40 μg/mL	PHMB 80 μg/mL	Cadmium	Cd + Inhibitor
Mean	3.62	0.33	9.06	37.77 ***	0.33
±					
SEM	3.29	0.25	6.2	9.18	0.23
**Caco-2 cells**	**Control**	**PHMB 40 μg/mL**	**PHMB 80 μg/mL**	**Cadmium**	**Cd + Inhibitor**
Mean	3.92	3.33	6.65	47.80 ***	3.33
±					
SEM	2.39	0.0	5.2	8.80	2.33
**Hep-G2**	**Control**	**PHMB 40 μg/mL**	**PHMB 80 μg/mL**	**Cadmium**	**Cd + Inhibitor**
Mean	3.22	0.53	3.06	87.21 ***	0.35
±					
SEM	2.89	0.37	3.2	9.28	0.09

Each value represents the mean ± SEM; n = 3. *** *p* < 0.001: significantly different as compared to control.

### 3.7. Effects of PHMB on the Expression of Select Genes Involved in Cell Cycle Regulation and Programmed Cell Death

Because Caco-2 cells do not have a functional *p53* gene, a second quantitative assay was performed with 3 times more material loaded in the gel. Only the concentration of 80 μg/mL has been tested for HepG2 cells. Treatment with PHMB had no effect on gene the expression of *bax*, *bcl-2*, *p21* or *p53* in any of the cell lines tested (see Table 8).

**Table 8:** Expression of genes involved in cell cycle and cell death in the presence of PHMB in Caco-2, Neuro-2A and HepG2 cells, as determined by RT-PCR. Because Caco-2 cells do not have a functional *p53* gene, a second quantitative assay was run in which 3 times the amount of DNA was loaded on the gel. Gene expression in HepG2 cells was investigated only at 80 μg/mL PHMB.

**Parameters**	**Caco-2**		**Neuro-2**		**HepG2**	**Control**
	**40** **μg/mL**	**80** **μg/mL**	**40** **μg/mL**	**80** **μg/mL**	**80** **μg/mL**	
**bcl-2**	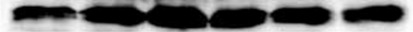					
**p53**	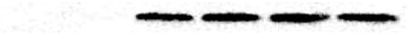					
**bax**	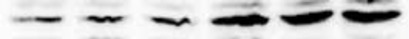					
**p21**	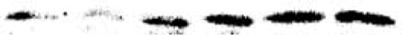					
**3 x (p53)**	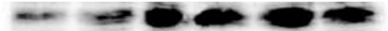					
**GAPDH**	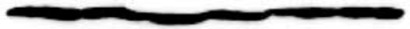					

Following evaluation of gene expression, the protein concentration of *bax*, *bcl-2*, *p21*, and *p53* were determined using their respective antibodies, mouse antibodies mostly reacting with the human antigens. Using anti-human *p53* did not result in larger amounts of the proteins measured (Table 9, Table 10 and Table 11). The relative values are ratio of amounts of each protein in treated cells/amounts of each protein in control cells. In control cells, the absolute values for *p21* and *bax* were in the range of 40–60 pg, and for *bcl-2* and *p53* in the range of 0.1–0.2 ng in l mg of protein extract tested.

**Table 9:** Relative values of *p53*, *p21*, *bax*, and *bcl-2* proteins in Neuro-2A cells incubated with PHMB for 24 h. Measurements were done using the Koma-ELISA kit (Japan). Plates were pretreated with anti-mouse antibodies. Values are expressed as mean ± SEM without unit.

Protein	PHMB 40 μg/mL	PHMB 80 μg/mL	Cd^2+^	Cd^2+^ + caspase inhibitor
p53	1.22 ± 0.2 **	1.12 ± 0.2 **	3.3 ± 0.1	1.02 ± 0.1
p21	1.05 ± 0.1 **	1.15 ± 0.1 **	3.55 ± 0.1	1.10 ± 0.1
bax	1.07 ± 0.05 **	1.17 ± 0.1 **	5.1 ± 0.5	1.07 ± 0.1
bcl-2	1.1 ± 0.1 **	1.2 ± 0.2 **	2.75 ± 0.5	1.1 ± 0.2

** *p* < 0.01; Cd^2+^ *vs.* PHMB.

**Table 10:** Relative values of *p53*, *p21*, *bax*, *bcl-2* proteins in Caco-2 cells incubated with PHMB for 24 h. Measurements were made using the Koma-ELISA kit (Japan). Plates were pretreated with anti-mouse antibodies. Values are expressed as mean ± SEM without unit.

Protein	PHMB 40 μg/mL	PHMB 80 μg/mL	Cd^2+^	Cd^2+^ + caspase inhibitor
p53	0.75 ± 0.1 **	0.72 ± 0.1 **	1.3 ± 0.3	1.12 ± 0.2
p21	0.65 ± 0.1 **	0.5 ± 0.1 **	1.55 ± 0.5	1.20 ± 0.2
bax	1.7 ± 0.75 **	1.25 ± 0.25 **	4.1 ± 0.3	1.7 ± 0.5
bcl-2	1.5 ± 0.5 **	1.2 ± 0.2 **	3.55 ± 0.5	1.5 ± 0.5

** *p* < 0.01; Cd^2+^ *vs.* PHMB.

**Table 11:** Relative values of of *p53*, *p21*, *bax*, *bcl-2* proteins in HepG2 incubated for 24 h. Measurements were made using the Koma-ELISA kit (Japan). Plates are pre treated by anti mouse antibodies that cross-react with the human antigen. Values are expressed as mean ± SEM without unit.

Protein	PHMB 40 μg/mL	PHMB 80 μg/mL	Cd^2+^	Cd^2+^ + caspase inhibitor
p53	1.33 ± 025 **	1.25 ± 0.2 **	5.3 ± 0.3	1.12 ± 0.1
p21	1.35 ± 0.3 **	1.25 ± 0.15 **	5.55 ± 0.5	1.20 ± 0.1
bax	1.55 ± 0.70 **	1.25 ± 0.25 **	4.1 ± 0.3	1.7 ± 0.5
bcl-2	1.75 ± 0.60 **	1.25 ± 0.25 **	3.55 ± 0.5	1.5 ± 0.5

** *p* < 0.01; Cd^2+^ *vs.* PHMB.

### 3.8. Effects on PHMB on IL-1 apha, TNF alpha and NF-κB

Three specific cytokines were determined and quantified by ELISA method using commercially available kits. These are interleukin 1 alpha, TNF alpha, (Tumor necrosing factor) and nuclear factor Kappa-B. All bear pro-inflammatory properties and display mitogenic activities [33]. They are produced in conditions involving oxidative stress and or necrosis followed by regeneration.

Interleukin 1 alpha, TNF alpha and Nuclear Factor Kappa B have been measured in the cellular homogenate, following 12 and/or 24 h incubation with and without PHMB (80 μg/mL) using commercial kits available. In order to evaluate their implication in possible cell proliferation following incubation with PHMB cells have been incubated for 12 h and 24 h, Cells were incubated with PHMB for 12 and 24 h, however, because PHMB-induced effects were slightly higher after treatment for 24 h compared to 12 h, only the results for the 24 h incubation are presented. Cytokine concentrations in the negative controls for each cell line were in the published range for cytokine production in these cell types and their tissue of origin (Table 12) [34]. Following treatment with 80 μg/mL PHMB, the production of IL-1 alpha, TNF alpha and NF-κB increased in all cell types (Table 13), however, the largest increases were seen in the HepG2 liver cell line where concentrations were 2–3 fold higher in the PHMB treated cells compared to the negative control (Figure 1 and Table 13).

**Table 12:** Amounts of cytokines and transcription factors in the control cells in the absence of PHMB, as measured using different commercially available kits.

Cytokines in Control cells (pg/mg of protein)	Neuro-2A	Caco-2	Hep-G2
Il-1 alpha	0.19	0.16	1.50
TNF alpha	0.08	0.05	0.70
NF-KB	0.96	0.08	1.08

The results for PHMB treated cells (12 h–24 h) are expressed as relatives values (ratio of concentration in treated cells to concentration in negative control cells; n = 4). Any value >1 is indicative of increased cytokines production. Although increases observed were low, increases in the concentration of all 3 analytes were significant in the HepG2 cells. Only the increase in TNF alpha concentration was significant in Caco-2 cells and only the increase in NF-κB was significant in Neuro-2A (Table 13).

**Table 13:** Quantification of (interleukin 1 alpha, TNF alpha and nuclear factor kappa B) in the cellular homogenate, following 24 h incubation with PHMB (80 μg/mL). The cytokines were quantified using different ELISA kits.

Cytokines	PHMB (80 μg/mL)		
	Neuro-2A	Caco-2	Hep-G2
Il-1 alpha	1.65 ± 0.20	1.35 ± 0.1	3.35 ± 0.2
TNF alpha	1.2 ± 0.25	1.95 ± 0.1	2.75 ± 0.2
NF-κB	1.8 ± 0.5	1.3 ± 0.1	2.5 ± 0.1

### 3.9. Effect of PHMB on GJIC

The integrity of the gap junctional intercellular communication has been evaluated by RT-PCR using oligonucleotide probe of the gene encoding connexin 43 (Table 1).

Cells were treated for 12 and 24 h, however, since the effects of PHMB were similar at all concentrations (20, 40 and 80 μg/mL) and for the two treatment intervals, only the data for treatment with 80 μg/mL PHMB for 24 h are presented (Table 14). The integrity of GJIC was evaluated also by measuring Connexin-43 by ELISA, using commercially available antibodies. The results are expressed in relative values (values of treated cells/value of control cells), and even though values are 1 or less, there is no apparent inhibition of GJIC by PHMB.

**Table 14:** Quantification by ELISA of Connexin 43 in the cellular homogenate following 24 h incubation with 80 μg/mL PHMB.

Parameter	PHMB (80 μg/mL)		
	Neuro-2A	Caco-2	HepG2
RT-PCR			
Connexin-43 Relative values	1.02 ± 0.15	0.95 ± 0.1	0.90 ± 0.25

## 4. Discussion

The existing cytotoxicity data were obtained from studies using either skin painting or administration of PHMB through the diet. The studies mainly focused on PHMB effects on the liver, but also examined the gut and brain since these are the target organs [8]. For these reasons the 3 cell types, Caco-2 cells (from a human colon adenocarcinoma) with a *p53* non-functional gene (∆p53: mut p53) and Neuro-2A (from a mouse neuroblastoma cells), and HepG2 cells (from a human hepatocellular carcinoma) with functional *p53* genes were selected.

During the determination of the PHMB IC 50% and evaluation of cell viability, two aspects of the effects on mammalian cells were noted. Firstly cytotoxic effects were observed rapidly less than 3 after the beginning or treatment with the human liver cells (HepG2) being the most sensitive. Secondly, when PHMB concentrations were very low, an hormesis-like effect was found, with about a 20% increase in cell proliferation, as reported by Creppy [35], however the studies reported here focused on the cytotoxic effects of PHMB which occur at much higher concentrations.

As measured by the MTT test, HepG2 cells are the most sensitive to the cytotoxic effects of PHMB among the three cell lines tested. This is in line with the fact that liver is regarded as the main target organ for PHMB in vivo [8] (US-EPA, 2004). Interestingly the neutral red test showed that the cell lines had a similar sensitivity to PHMB at much lower concentrations than with the MTT assay, thus indicating that the cellular target is the membrane. This is the reason why all of the cells blow up when the respective cytotoxic concentrations for PHMB are reached in the culture medium. It is interesting to note that PHMB has a similar effect on bacteria [2,36]. Because of the structural similarities between antimicrobial peptides (AMPs) and PHMB, the latter can be incorporated into bacterial cell membranes and kill bacteria in a manner similar to AMPs [2]. This mechanism of action is quick and means that bacteria are unlikely to develop resistance to PHMB [36]. AMPs, which are found in all kingdoms, function primarily by permeabilizing the bacterial membrane. AMPs have several advantages over existing antibiotics including their rapid bactericidal activity, and PHMB bears very similar properties. The little data available for mammalian cells are similar to the present finding [1,3].

Similar to viability results using the neutral red test, cytotoxicity testing using LDH leakage into the culture medium also showed that PHMB is cytotoxic to the three cell lines studied, however, the HepG2 human liver cell line is again the most sensitive to the effects of PHMB. The cytotoxicity targeting the cell membrane occurs rapidly with a statistically significant increase in LDH leakage observed after 3 h exposure to PHMB at concentrations of 20 μg/mL, and maximum leakage at 40 to 80 μg/mL. At these levels of LDH leakage, 50% of HepG2 cells were killed whereas the LC 50% for the Caco-2 and Neuro-2A cells was 160 μg of PHMB/mL.

DNA fragmentation examined by agarose gel electrophoresis showed that there was no significant increase in DNA fragments following exposure to PHMB as would be expected if the cells were undergoing apoptosis, and results of the comet test, confirm that PHMB is not genotoxic (clastogenic).

Oxidative stress, a common cause of epigenetic changes to DNA [33] was evaluated by quantifying the levels of MDA and 8-OHdG in PHMB treated cells. PHMB did not induce an increase in MDA concentration and DNA bases oxidation was not significantly increased (approximately 10%) compared to the negative control confirming the very low rate of oxidative stress. DNA methylation and histone modifications are two common epigenetic mechanisms that cells use to regulate replication [16,17,18,20,29,31,32,33,37,38] therefore, determination of the DNA methylation rate is necessary when dealing with a compound with possible epigenetic effects. In the case of PHMB, DNA cytosine methylation rate increased slightly (15–20%) but was not concentration dependent, occurring mainly in intestinal and hepatic cells and not in neuronal cells. The DNA methylation rate is generally higher in brain cells than in intestinal cells and in hepatocytes [20,32,33,38].

In addition to the DNA methylation rate, cytokine activity also has an influence on cell division and together, these parameters are determinant for overall cellular proliferation. Therefore, cytokine and transcription factor production (interleukin 1 alpha, TNF alpha and nuclear factor kappa B) were evaluated in the cells types studied. HepG2 cells, originating from liver, showed a higher production of cytokines (2–3 fold) compared to the control cells at 80 μg of PHMB, which is the IC 50% for this cell type in viability test, while it represents only half of the IC 50% value for Caco-2 and Neuro-2A cell types (Figure 1 and Table 13). The increase of these cytokines, although low, was significant in HepG2 cells, and there was a weak but significant increase in TNF alpha and in NF-κB in Caco-2 cells, and in Neuro-2A cells, respectively. The mitogenic effect of these cytokines could be enhanced if there was an interruption in the gap junction intercellular communication allowing cells to escape from contact inhibition [16,29]. However, there is apparently no inhibition of the GJIC by PHMB at concentrations in the range of the IC 50% values in any of the cell lines studies.

The mechanism by which PHMB kills mammalian cells is still a matter of investigation. It does appear that the membrane is the target resulting in cell lysis and cell death, just as in bacteria [3,39]. Röhner *et al.* reported that lactate dehydrogenase activity showed a significant increase in the culture medium of human chondrocytes after a short incubation time with PHMB [39], confirming the data in our studies and indicating that necrosis might occur in tissues followed by inflammation. However it is possible that PHMB exposed cells may undergo an apoptotic cell death, therefore, we also investigated several mechanisms leading to apoptosis and cell death.

There was very weak or no activation of caspase-3 in the any of the cells lines treated with PHMB indicating that the cells are not undergoing apoptosis. Choy *et al.* very recently reported on the human corneal epithelial cells exposure to (PHMB) (0.00015%) (MPS-A), PHMB (0.0005%) (MPS-B) and PHMB (0.0001%) (MPS-C) diluted to 10–40%, for 1, 5 and 10 min [4]. Cell viability and membrane integrity were assessed by MTT test flow cytometry following staining with AnnexinV-FITC/7-AAD. Their data showed that after 10 minutes exposure, almost 40 per cent of cells in MPS-A but less than five per cent in MPS-B or MPS-C, were in late necrotic stage. No apoptosis was observed. After 12 h of exposure, cell activity was significantly reduced in a concentration dependent manner for MPS-A treated cells only (*p* > 0.05).

Although caspase-3 was not activated it is possible that other caspases such as caspase-1 which induces cell death by pyroptosis, were activated. Pyroptosis is a caspase-1-dependent programmed cell death, which features rapid plasma membrane rupture, DNA fragmentation, and release of proinflammatory intracellular contents [40,41]. This caspase 1-dependent cell death, is inherently inflammatory, is triggered by various pathological stimuli, such as stroke, heart attack or cancer, and is crucial for controlling microbial infections [42]. Caspase-1 plays a key role in host defense through its dual functions in inducing a pro-inflammatory cell death and in promoting the secretion of pro-inflammatory cytokines, and a new study highlighted the specific importance of pyroptosis in resistance to intracellular pathogens [41,42,43]. However, since our data do not show no indication of DNA cleavage, and since pyroptosis is mainly related to an inflammatory host response, it does not appear that caspase 1-dependent cell death occurred in the PHMB treated cell lines we investigated.

Additionally effectors in programmed cell death, such as *p53*, *p21*, *bax*, and *bcl-2* did not show any increase in gene expression or at the protein level, therefore, it does not appear that PHMB induced cell death is not the result of apoptotsis, but rather is cytotoxic at the cell membrane level resulting in necrotic cell death.

At concentrations of 1 to 100–200 μg/mL, the effects of PHMB are weak only occurring at cytotoxic concentrations and are not concentration-dependent. Our proposed mechanism of action for PHMB induced cell death is as follows: at very low concentrations, 0.1 to 0.5–1 μg/mL, PHMB binds to the cell membrane facilitating uptake of nutrients and ion channel trafficking, resulting in increased cell viability and division (hormesis effect). However, when PHMB is administered at higher concentrations (>20–50 μg in culture medium) which correspond to 20–100 and up to 500 mg/L in vivo, the increased binding of PHMB to the membrane is cytotoxic resulting in lysis of the membrane with leakage of enzymes and cytokines and eventually cell death by necrosis.

## 5. Conclusions

Given that PHMB is non-genotoxic and non-mutagenic, studies were undertaken to evaluate possible epigenetic properties of PHMB. The present data obtained in vitro demonstrate that even at high concentrations, PHMB induces only a weak production of some cytokines and the transcription factor NF-κB and does not explain the occurrence of hepatocyte tumors observed in rodents. The observed rodent tumors occurred only at high doses (1000 and 1500 mg/L; NOEL 500 mg/L) in drinking water well above those to which humans will ever be exposed. However, until the mechanism for PHMB induced tumors has been elucidated, their relevancy to human risk assessment, cannot be determined. Research to this end is currently underway.
